# Mitigation of bacterial spot disease induced biotic stress in *Capsicum annuum* L. cultivars via antioxidant enzymes and isoforms

**DOI:** 10.1038/s41598-021-88797-1

**Published:** 2021-05-03

**Authors:** Musarrat Ramzan, Sundas Sana, Nida Javaid, Anis Ali Shah, Samina Ejaz, Waqas Nazir Malik, Nasim Ahmad Yasin, Saud Alamri, Manzer H. Siddiqui, Rahul Datta, Shah Fahad, Nazia Tahir, Sidra Mubeen, Niaz Ahmed, Muhammad Arif Ali, Ayman El Sabagh, Subhan Danish

**Affiliations:** 1 Department of Botany, The Islamia University of Bahawalpur, Bahawalpur, 63100 Pakistan; 2 Department of Botany, University of Narowal, Narowal, 51801 Pakistan; 3 Department of Biochemistry and Biotechnology, The Islamia University of Bahawalpur, Bahawalpur, Pakistan; 4 Senior Superintendent Gardens, Resident Officer-II Office Department, University of the Punjab, Lahore, 54590 Pakistan; 5 Department of Botany and Microbiology, College of Science, King Saud University, Riyadh, 2455 Saudi Arabia; 6 Department of Geology and Pedology, Faculty of Forestry and Wood Technology, Mendel University in Brno, Zemedelska 3, 61300 Brno, Czech Republic; 7 Hainan Key Laboratory for Sustainable Utilization of Tropical Bioresource, College of Tropical Crops, Hainan University, Haikou, 570228 China; 8 Department of Agronomy, The University of Haripur, Haripur, 22620 Pakistan; 9 Department of Agriculture, AbdulWali Khan University, Mardan, Pakistan; 10 Institute of Agriculture Resource and Regional Planning, Graduate School of Chinese Academy of Agriculture Sciences China, Changchun, China; 11 Department of Chemistry, The Women University Multan, Punjab, 60800 Pakistan; 12 Department of Soil Science, Faculty of Agricultural Sciences and Technology, Bahauddin Zakariya University, Multan, 60800 Pakistan; 13 Department of Agronomy, Faculty of Agriculture, University of Kafrelsheikh, Kafr El-Shaikh, Egypt

**Keywords:** Plant sciences, Plant stress responses, Biotic

## Abstract

Bacterial spot, caused by a group of *Xanthomonads* (*Xanthomonas* spp.), is a devastating disease. It can adversely affect the *Capsicum annum* productivity. Scientists are working on the role of antioxidants to meet this challenge. However, research is lacking on the role of antioxidant enzymes and their isoforms in the non-compatible pathogen and host plant interaction and resistance mechanisms in *capsicum* varieties. The present study was conducted to ascertain the defensive role of antioxidant enzymes and their isoforms in chilli varieties Hybrid, Desi, Serrano, Padron, and Shehzadi against bacterial spot disease-induced *Xanthomonas* sp. The seedlings were inoculated with bacterial pathogen @ 10^7^ CFU/mL, and samples were harvested after regular intervals of 24 h for 4 days followed by inoculation. Total plant proteins were extracted in phosphate buffer and quantified through Bradford assay. The crude protein extracts were analyzed through quantitative enzymatic assays in order to document activity levels of various antioxidant enzymes, including peroxidase (POD), Catalase (CAT), Ascorbate peroxidase (APX), and Superoxide dismutase (SOD). Moreover, the profiles appearance of these enzymes and their isoforms were determined using native polyacrylamide gel electrophoresis (PAGE) analysis. These enzymes exhibited maximum activity in Hybrid (HiR) cultivar followed by Desi (R), Serrano (S), Padron, and Shehzadi (HS). Both the number of isoforms and expression levels were higher in highly resistant cultivars compared to susceptible and highly susceptible cultivars. The induction of POD, CAT, and SOD occurs at the early stages of growth in resistant *Capsicum* cultivars. At the same time, APX seems to make the second line of antioxidant defense mechanisms. We found that modulating antioxidant enzymes and isoforms activity at the seedling stage was an important mechanism for mitigating plant growth inhibition in the resistant ones.

## Introduction

Pepper and sweet pepper are important economically valuable crops around/throughout the world^1^. Their fruits are extremely nutritious as they have remarkable macronutrients such as carbohydrates, proteins, dietary fibers and fats^2,3^.

However, its growth and production are influenced by complex abiotic^4–10,11–13^ and biotic stresses that induce unfavorable changes at the cellular and molecular levels^14–16^. One of the most harmful pepper crop diseases is “bacterial spot”, caused by *Xanthomonas* spp. Plant and pathogen contacts are arbitrated through a network of cytological and molecular events generating a continuum of resistance and susceptibility^17^. *Xanthomonas* species are widespread, presumably because of contaminated seeds^18^. The bacterial spot’s symptoms appear on the host leaf between 5 and 8 days after *X. campestris* inoculation^19^. Initial bacterial spot disease symptoms are water-soaked circular lesions that later turned dark brown to black^20^. Pathogen attacks cause cellular homeostasis disruption by reactive oxygen species (ROS) production^21^.

Like animals and humans, plants can not more from one place to another to protect them from adversely affecting situations. For protection, especially against the pathogen, plants reply on innate immune mechanisms as a defensive tool^22^. The antioxidant system is a very efficient system evolved by plants to cope up supra-optimally produced superoxide radicals, its derivative free radicals and other organic radicals^20–25^. The first discovered and characterized antioxidant enzyme is Catalase (CAT). Catalase scavenges hydrogen peroxide (H_2_O_2_) in peroxisomes whereas Ascorbate peroxidase uses ascorbic acid (AA) as a reducing agent which reduces H_2_O_2_ to H_2_O^26^. A rise in the activity level of antioxidant enzymes reduces the extent of oxidative damage and restores photosynthetic imbalance caused by the production of bacterial lesions. Peroxidase (POD) is responsible for regulating plant metabolism, photosynthesis, respiration, substratum oxidation, cell wall lignification and growth, and infections^27^. Superoxide dismutases (SODs) are omnipresent and function by catalyzing the irregular superoxide anions (O_2_^−^)^28^. Kuznaik and Sklodowska^29^ analyzed peroxisomal antioxidant enzymes viz. SOD, CAT, Glutathione peroxidase (GPX), and ascorbate–glutathione (AA-GSH) cycle activities in tomato infected with *B. cinerea.* It was early noticed increases in SOD, CAT and GPX indicating that initial infection induced their activities and consequently the activation of antioxidant plant defense, which was followed by a progressive inhibition concomitant with disease symptoms development. Depending upon the severity of pathogenic activity and its interaction with host, scavenging potential of antioxidants i.e., SOD, CAT and peroxidase (POX) produced by host also become variable^30^. Furthermore, the activities of AA-GSH cycle enzymes APX, monodehydroascorbate (MDHAR), dehydroascorbate (DHAR) and glutathione reductase (GR) as well as ascorbate and glutathione concentrations and redox reactions were significantly decreased with the differential rate and timing of activity. However, research is lacking on the role of antioxidant enzymes and their isoforms in the non-compatible pathogen and host plant interaction and resistance mechanisms in *capsicum* varieties.

This study’s primary goal was to differentiate *capsicum* varieties based on their ability to cope with the stress induced by the bacterial spot pathogen. The study was further extended to get an insight into the mechanistic details of the defense mechanisms. Thus, in the current study, we investigated the role of different antioxidant enzymes (SOD, POD, CAT, and APX) in the defense mechanism of *capsicum* cultivars, enabling them to mitigate the infection damage on them. It is hypothesized that antioxidant enzymes (SOD, POD, CAT, and APX) could be evolved as a defense of *capsicum* mechanism against the bacterial spot pathogen during their coevolution process in the past time.

## Results

Screening of five chilli cultivars for bacterial spot disease was performed. Disease score different levels were observed ranging 0–40%. The chilli pepper cultivar Hybrid (HiR) was completely free from bacterial spot disease. Desi pepper was found to be resistant, and Serrano pepper was considered susceptible. Two cultivars (Padron and Shehzadi) were susceptible to bacterial spot disease (Table 1).

**Table 1:** Bacterial spot severity on five chilli cultivars challenged by a Xanthomonas spp., bacterial spot disease agent.

S No	Varieties	Bacterial spot incidence (%)	Categorization
1	Hybrid	0	HiR
2	Desi	6	R
3	Serrano	22	S
4	Padron	33	HS
5	Shehzadi	40	HS

Screening results of five *Capsicum* varieties under bacterial spot disease stress in greenhouse conditions. Chilli plants were grown in plastic pots, and after 21 days of growth, seedlings were inoculated with *Xanthomonas campestris*.

Bacterial spot disease incidence was observed up to 60 days from the day of bacterial inoculation.

*HIR* highly resistant, *R* resistant, *S* susceptible, and *HS* highly susceptible.

### H_2_O_2_ and MDA contents

The plants inoculated with *X. campestris* pathogen showed an increased H_2_O_2_ and MDA contents in chilli seedlings after 72 h of inoculation than non-treated plants (Table 2). In contrast, MDA and H_2_O_2_ contents were increased in Shehzadi followed by Padron, whereas Hybrid exhibited the least H_2_O_2_ and MDA contents.

**Table 2:** The oxidative stress parameters values (mean ± SE) of chilli varieties against bacterial spot disease recorded after 72 h of inoculation.

Varieties	Treatment	H_2_O_2_ content (µmol mg^−1^ protein)	MDA content (µmol mg^−1^ protein)
Hybrid	Control	10 ± 0.314 g	0.98 ± 0.005 g
	Treated	10.2 ± 0.20 f.	1.2 ± 0.033 e
Desi	Control	10.3 ± 0.086 f.	1.4 ± 0.033 de
	Treated	11.1 ± 0.04 e	1.7 ± 0.033 c
Serrano	Control	13.5 ± 0.085 d	1.8 ± 0.033 c
	Treated	19.5 ± 0.06 c	2.8 ± 0.05 b
Padron	Control	12.3 ± 0.091 d	1.6 ± 0.033 d
	Treated	24.8 ± 0.173 ab	3.4 ± 0.057 a
Shehzadi	Control	11.7 ± 0.033 e	1.0 ± 0.057 f.
	Treated	25.2 ± 0.088 a	3.5 ± 0.033 a

Values demonstrate means ± SE (n = 5).

Different letters indicate a significant difference among the treatment (p < 0.05).

### Temporal changes in POD activity

**Figure 1:**
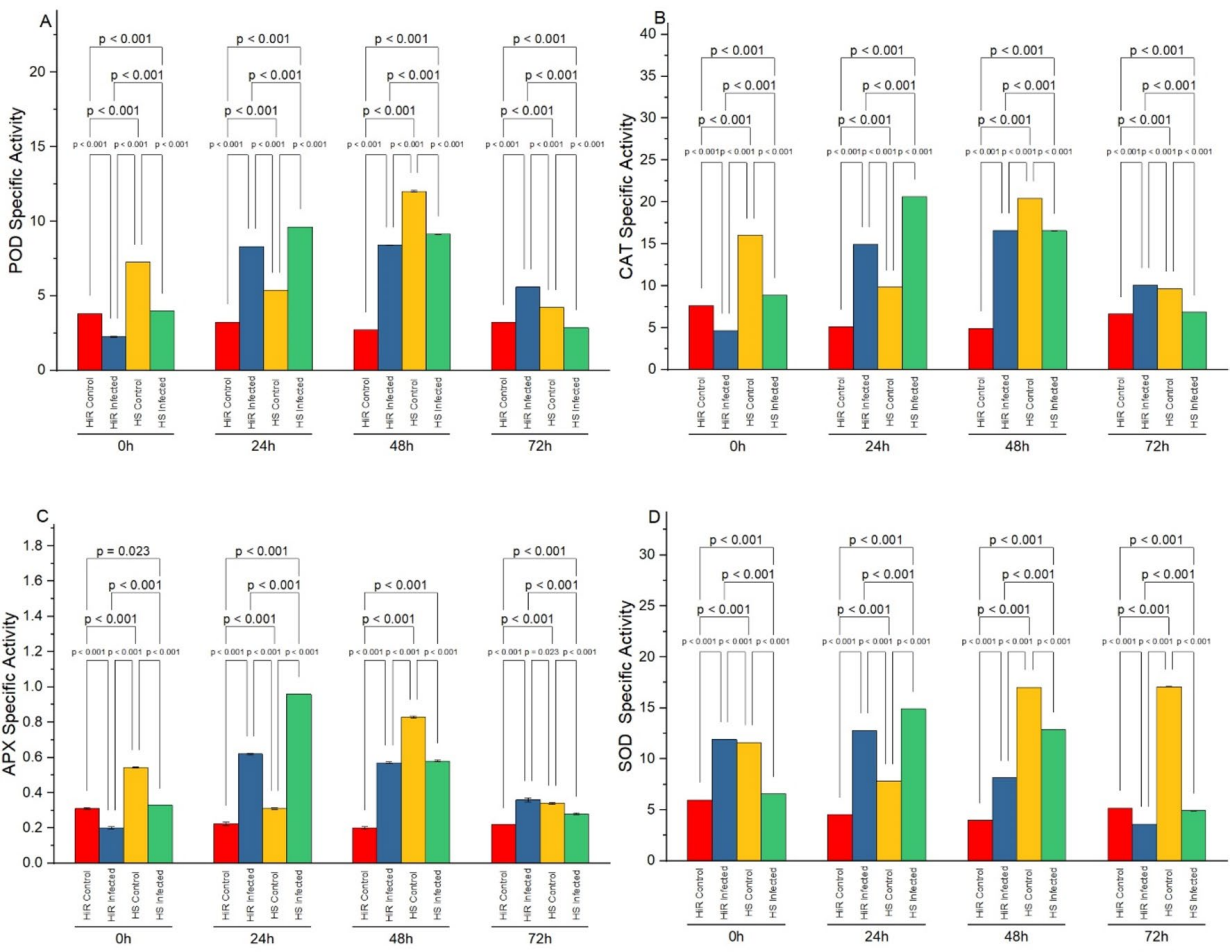
Specific activity of antioxidant (**A**) POD (U mg^1^ Proteinmin^1^) activity, (**B**) CAT (U mg^1^ Proteinmin^1^) activity, (**C**) APX (U mg^1^ Proteinmin^1^) activity, and (**D**) SOD (U mg^1^ Proteinmin^1^) activity in highly resistant (Hybrid) and highly susceptible (Padron) cultivars with and without *Xanthomonas* inoculation. Bar values indicates p-values at α < 0.05.

**Figure 2:**
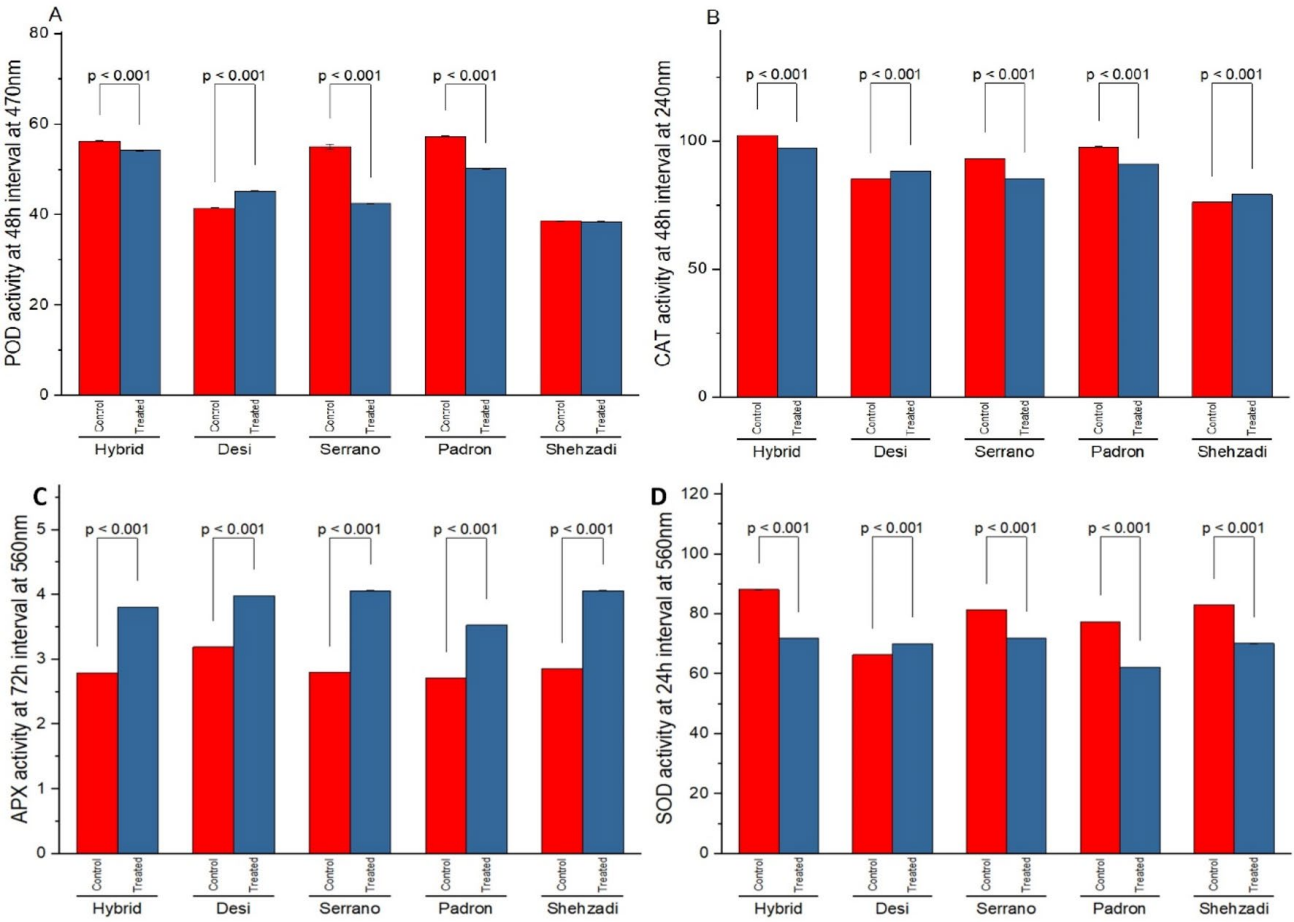
Antioxidant activity in Hybrid, Desi, Serrano, Padron, and Shehzadi chilli cultivars: (**A,B**) POD (U mg^1^ FW) and CAT (U mg^1^ FW) activity at 48 h, (**C**) APX activity at 72 h, and (**D**) SOD (U mg^1^ FW) activity at 24 h with inoculation of the pathogen. Data is an average of 3 independent experiments following 3 replicates. Bar values indicates p-values at α < 0.05.

The temporal changes in Peroxidase activity of highly resistant (HiR) Hybrid and highly susceptible (HS) Padron seedlings with and without pathogen are shown in Figs. 1 and 2. Varying patterns of POD (Fig. 1A) CAT (Fig. 1B), APX (Fig. 1C), and SOD (Fig. 1D) activities were observed in control and inoculated seedlings of HS and HiR varieties. A steady enhancement in POD activity was observed in all varieties. In resistant seedlings, a drastic enhancement in POD activity was observed initially after inoculation of the pathogen, which reached the peak at 48 h.p.i (54.2 min^−1^ g^−1^ FW) whereas, in highly susceptible seedling, POD activity was found to be 50.2 min^−1^ g^−1^ FW at 48 h.p.i. after inoculation (Fig. 2A). Specific activities of CAT (Fig. 2B) and APX (Fig. 2C) show almost the same behavior during temporal phases of inoculation except for SOD (Fig. 2D) at 0 h and 72 h.

### Native PAGE analysis of POD

**Figure 3:**
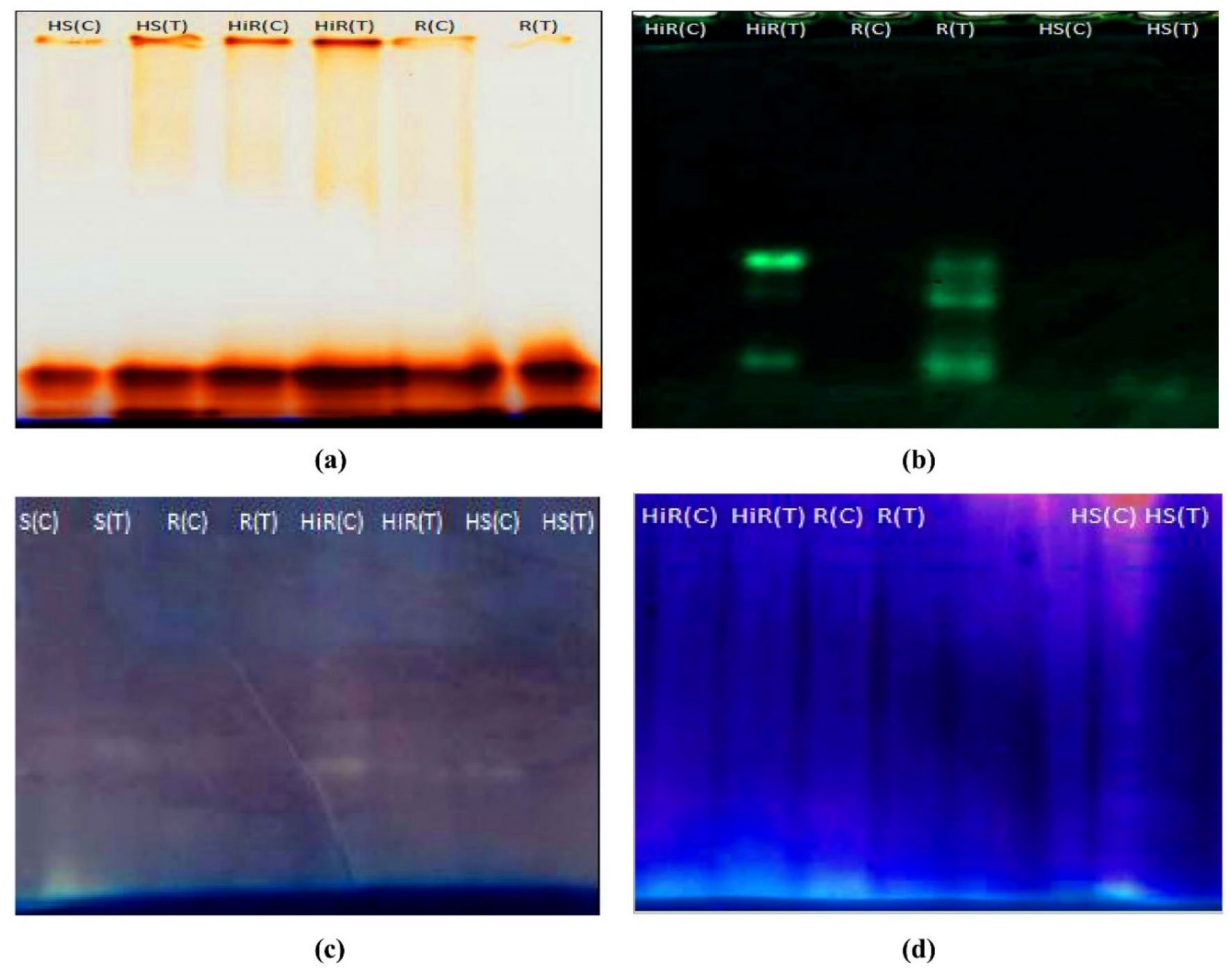
Gel activity detection, (**a**) POD activity gel image, (**b**) CAT activity gel image, (**c**) SOD activity gel image, (**d**) APX activity gel image in HiR, R, S and HS chili varieties in the absence and presence of *X.* spp. The sample loading amount used in each line was 80 µg protein. *R* resistant, *HiR* highly resistant, *S* susceptible, *HS* highly susceptible, *C* control, *T* treatment (infected).

**Figure 4:**
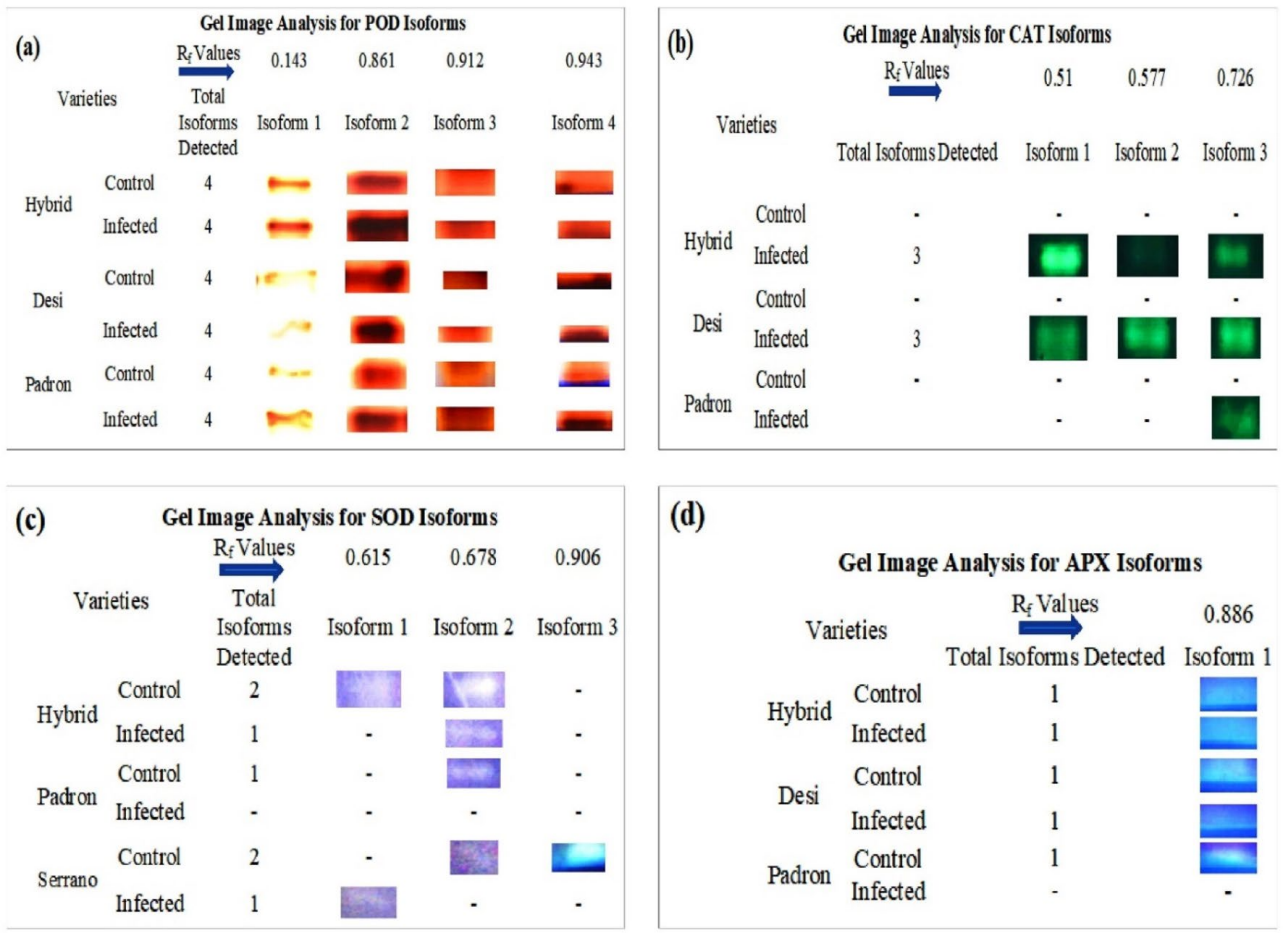
Gel image analysis of isoforms of POD (**a**), CAT (**b**), SOD (**c**), and APX (**d**). Four isoforms were detected in both control and infected plants of the cultivars Hybrid, Desi and Padron. An infected plant of the cultivar Hybrid showed the higher expression of all four isoforms of POD enzyme; (**b**) CAT activity was not detected on the gel in control samples of all cultivars. A Total of three isoforms were detected in infected plants of “Hybrid” and “Desi”. Infected plant of “Padron” did not show any expression of isoform 1 and 2. (**c**) A total of three isoforms were expressed; isoform 1 was expressed in the control plant (cv.Hybrid) and infected plant (cv. Serrano). Isoform 2 was expressed in all plant samples, except the controls, of the less resistant cultivars (Padron and Serrano), whereas isoform 3 was detected only in the control sample of “Serrano” and (**d**) only one isoform was detected of APX in the cultivars Hybrid and Desi, but it was not detected in the infected plant of cv. Padron.

The POD isoforms detection where analyzed with the total protein samples of HS and HiR, cultivars. A total of four isoforms appeared, and their strength varied between control and inoculated seedlings. Infected plants have an expression of more POD isoforms as compared to their respective controls. Infected HS and HiR cultivar had an equal number of isoforms (four), whereas the non -inoculated plant has three isoforms. POD isoform’s protein bands were low in intensity in HS over HiR seedlings (Figs. 3, 4).

### CAT activity temporal changes

The temporal fluctuations in CAT activity of HS and HiR inoculated, non-inoculated seedlings are shown in Figs. 1B and 2B. A continuous increase in CAT activity was noticed for all cultivars. In highly resistant chilli seedling, a drastic increase in CAT activity was observed after pathogen inoculation and reached its peak at 48 h (102.3 min^−1^ g^−1^ FW) whereas, in HS seedlings, CAT activity was observed to be 91.1 min^−1^ g^−1^ FW (Fig. 2) at 48 h after inoculation of the pathogen.

### Native PAGE for CAT

Protein samples of HS and HiR *Capsicum* seedlings were examined for the appearance of CAT isoforms. A total of 3 isoforms of CAT were appeared. Expression of CAT isoforms was low in HS seedlings as compared to HiR seedlings (Fig. 3b). Infected Hybrid pepper (HiR) has 3 CAT isoforms, whereas Padron pepper (HS) exhibits only 1 CAT isoform expression under *Xanthomonas campestris* treatment. Control chilli pepper variety has not shown any CAT isoform.

### Temporal changes in SOD activity

Figures 1D and 2D shows variable patterns of SOD activity in control and inoculated seedlings of HS and HiR varieties. In HiR, after pathogen inoculation, a noticeable enhancement in SOD activity was observed that reached the peak at 48 h (78 min^−1^ g^−1^ FW) whereas, in HS (Padron) seedlings, SOD activity was found to be 70 min^−1^ g^−1^ FW at 48 h after pathogen inoculation.

### Native PAGE analysis of SOD

SOD expression was not detected in highly resistant (Hybrid) variety in case of both inoculated and un-inoculated. Three isoforms of SOD were detected in control seedlings of resistant variety, while only one isoform was expressed in seedlings of treated resistant variety. Susceptible variety showed only one isoform expression, but the expression level was low in control seedlings compared to treated seedlings. Control chilli pepper (Padron) has shown only one isoform in gel detection assay (Figs. 3c, 4c).

### Temporal changes in APX activity

A continuous increase in APX activity was noticed for all cultivars. In HiR seedlings, after inoculation of pathogen, a significant increase in APX activity was noticed and reached at its peak (3.822 min^−1^ g^−1^ FW) at 0 h. while cv. Padron pepper (HS) had (3.99 min^−1^ g^−1^ FW) it at 24 h after inoculation (Figs. 1C, 2C).

### Native PAGE analysis of APX

A single isoform of APX was detected, and its strength varied between inoculated and control seedlings. The APX detection was low in intensity in HS seedlings over HiR seedlings (Figs. 3d, 4d). APX expression was not detected in treated Padron pepper (HS) may be due to low activity. Infected Hybrid (HiR) variety showed high expression of APX among other cultivars.

### Validation of POD, CAT, SOD and APX for five chilli cultivars

Seedlings of five different chilli cultivars were analyzed for POD, CAT, SOD and APX with and without bacterial inoculation. All the chilli cultivars showed an enhanced level of enzyme activity after inoculation. The highest activity of Peroxidase (54.2 min^−1^ g^−1^ FW) at 48 h.p.i was observed in cv. Hybrid (HiR) and lowest (50.2 min^−1^ g^−1^ FW) by cv. Padron (HS) after pathogen inoculation (Fig. 2A). Significantly highest CAT activity was found in cv. Hybrid (102.3 min^−1^ g^−1^ FW) after inoculation, over control and other cultivars and least by cv. Padron (91.1 min^−1^ g^−1^ FW) (Fig. 2B). SOD activity was found maximum (78.8 min^−1^ g^−1^ FW) in HiR and least in HS (70.8 min^−1^ g^−1^ FW) after inoculation (Fig. 2D). APX activity results differently than other studied antioxidant enzymes in chilli pepper plants against bacterial spot disease (Fig. 2C). Maximum activity was observed in cv. Desi (R) (4.312 min^−1^ g^−1^ FW) at 0 h followed by cv. Serrano, Shehzadi, Padron, and least activity was noticed in cv. Hybrid (HiR) (3.822 min^−1^ g^−1^ FW).

## Discussion

*Capsicum annuum* L. is a herbaceous biennial of the Solanaceae family that a quarter of the world’s population eats daily^31^. When challenged by pathogens, the plant’s first-line antioxidants include superoxide dismutase, catalase, and glutathione peroxidase becomes active in the whole defensive strategy^31^. In the present research, attempts have been made to explore the susceptible and resistance differences among chilli pepper cultivars to *Xanthomonas sp.* Infection and bacterial spot severity by considering POD, CAT, SOD, and APX as biochemical host resistant markers. The cultivars showed varying degrees of resistance and susceptibility to bacterial spot, as expected. Highly resistant (cv. Hybrid) expressed the maximum intensity of antioxidant enzymes compared with resistant (cv. Desi), susceptible (cv. Serrano), and highly susceptible (cvs. Padron and Shehzadi). Such results are well associated with observations of Kavitha and Umesha^27^, who also reported the relation of antioxidants production and tomato plant defense to the bacterial spot pathogen.

The early and rapid increase of various antioxidants is an important feature of plant-resistance to pathogenic agents^32^. In the present study, peroxidase activity in chilli significantly increased after pathogen inoculation. POD activity was maximum in cv. Hybrid (HiR) at 48 h after pathogen inoculation compared to control and least in Shehzadi pepper at 24 h. Such observations are consistent with Xie et al.^33^; their results revealed that POD activity increased in Patchouli against *Ralstonia solanacearum* the causal agent of bacterial wilt disease on solanaceous crops. Peroxidase native PAGE results showed that treated plants had increased POD isoforms compared to untreated plants. Padron (S) seedling isoforms had a low expression when compared to the cv. Hybrid (HiR) seedlings. In chilli infected by *Colletotrichum* and brinjal (eggplant-USA or aubergine-UK) by *Ralstonia solanacearum* exhibited dense and dark isoform in resistant compared to susceptible plants^34,35^.

After bacterial inoculation, CAT showed significant activity. In highly resistant cv. Hybrid at 48 h.p.i, CAT temporal changes were highest whereas least CAT activity was observed at 48 h.p.i. in treated cv. Padron pepper. However, Debona et al.^36^ revealed that the leaves of a resistant *P. oryzae*-infected wheat cultivar produced more CAT than susceptible cultivar. CAT detection on Native PAGE further confirmed the results of the spectrophotometric estimation. Hybrid (HiR) and Desi (R) *Capsicum* cultivars express three CAT isoforms upon bacterial inoculation, whereas infected cv. Padron (HS) had an expression of just one isoform.

Spectrophotometric SOD assay showed maximum activity in cv. Hybrid (HiR) after 48 h of post*-*inoculation of pathogen compared to susceptible cultivars. POD and SOD enzymatic activities increased significantly in patchouli inoculated with *R. solanacearum*^34^, and the findings of them and those of the present study were consistent with Chai et al.^37^ studies of antioxidant isozymes to some extent. Lobna et al.^38^ reported that POX, CAT, PPO, and SOD enzyme activity after pathogen infection increased in resistant cultivar compared to a susceptible one. The detection of SOD isoforms by PAGE showed the expression of two isoforms in control and one in infected S (Serrano) and R (Desi) variety, according to the results of Xie et al.^33^.

Rojas-Beltran et al.^39^ revealed that the chloroplast of most angiosperms has an expression of two SOD isoforms. A similar number of isoforms were detected in resistant and susceptible *Capsicum* cultivars as reported by García-Limones et al.^40^. APX enzyme activity was maximum in resistant (cv. Desi) and least in susceptible cultivars. Vuleta et al.^41^ have shown that the APX in the *Iris pumila* leaves was the main H_2_O_2_ scavenger when exposed to high light intensities. In the present investigation, just one type of APX isoform was observed in all cultivars. Chandrashekar and Umesha^17^, found similar results. During the bacterial spot pathogen attack, all four antioxidant enzymes (POD, CAT, SOD, and APX) participated in ROS-scavenging metabolism in chilli pepper varieties, in agreement with the results of^27^.

## Materials and methods

The experiments were done in a greenhouse at the Department of Botany, The Islamia University of Bahawalpur (IUB), Pakistan (29.37 latitude and 71.75 longitude) in consecutive three trials. River soil, peat moss, and sandy loam 39 were mixed (50:20:30%) and utilized at 30-35 °C and 61% (humidity) as growing medium. For 15-20 days soil was exposed to sun and placed in plastic pots (9 cm diameter). ^42^. Seeds were collected from the regional vegetable research agriculture institute of Bahawalpur, and surface-sterilized with sodium hypochlorite solution (0.1 %). After that 3 washing were given with sterilized water ^43^. Seeds were sown in the growing medium in pots. Twenty-one days old seedlings of chilli pepper cultivars Hybrid, Desi, Serrano, Padron, and Shehzadi were thinned for maintaining 3 healthy seedlings in each pot. Pots were arranged in a completely randomized design. Un-inoculated pots (n = 5) were used as control. The bacterial inoculum level used was at approximately 10^–7^/mL. The inoculation was carried out after 2 days of seedling thinning. The bacterial spot infected chili leaves were collected from greenhouse of Department of Botany, IUB. The seedlings were harvested at the time of inoculation (0 h) and 24 h intervals up to 72 h, initial inoculation. For bacterial source and growth in the laboratory, an ooze test was performed by cutting 1 cm of the infected portion of the plant, sterilized gently squeezed to get a suspension in a sterile test tube containing 3 mL sterilized saline. After dilution, it is plated on YDC Agar medium. After 24 h, incubation light yellow color colonies were developed resembling *Xanthomonas* sp. biochemical assay was carried out to confirm *X. campestris* species. Recovered colonies were harvested and suspended in flasks containing sterilized distilled water and incubated for 2–4 h at 32 ± 1 °C. Inoculum suspension was adjusted with a spectrophotometer. Plants were observed daily for bacterial spot symptoms. Depending on disease severity, the cultivars were divided into highly resistant (0%), resistant (0.1% to 10%), slight marginal spot, and 1–20% of leaves become brown, susceptible (10.1% to 20%) of plants showing sectorial spots and 20–40% of leaves become brown and highly susceptible (> 25%) of the plants showing leaf collapse and more than 40% of leaves become brown^17^.

### Determination of oxidative stress

H_2_O_2_ and Malondialdehyde (MDA) contents of chloroplast were examined by a previously described method of Diao et al.^44^.

### Temporal study of antioxidant enzymes

The antioxidant enzyme activity studied were POD, CAT, SOD, and APX, which were analyzed using the highly resistant (cv. Hybrid) and highly susceptible (cv. Padron), which were selected based on their performance on the pathogenicity tests carried out previously. This study aimed to find out the early response of antioxidant enzymes and their isoforms in resistance mechanism; for this purpose the chilli pepper seedlings were grown as previously enlightened, and seedlings were harvested at 0, 24, 48, and 72 h after *Xanthomonas* pathogen inoculation. Distilled water inoculated samples were used as a negative control. One gram of each chilli seedling was homogenized in 1 mL of 50 mM buffer (potassium-phosphate) of pH 7. The homogenate was centrifuged at 12,000 rpm for 15 min at 4 °C.

### Peroxidase assay

POD activity was determined according to Chance and Maehly^45^ method by monitoring the absorbance at 470 nm every 20 s. The activity was measured by H_2_O_2_ peroxidation with an electron donor (guaiacol) and the formation of tetra guaiacol. The reaction mixture consisted of 50 mM potassium phosphate buffer pH 5, 20 mM guaiacol, 40 mM H_2_O_2_ in 0.1 mL of sample enzyme extract (Supplementary Data 1).

### Catalase assay

CAT activity was analyzed by following the method described by Chance and Maehly^45^. The reaction mixture (3 mL) containing 50 mM phosphate buffer (pH 0.0), 5.9 mM H_2_O_2,_ and 0.1 mL enzyme extract was assayed. The CAT activity was measured by a change in absorbance due to H_2_O_2_ intake at 240 nm after every 20 s.

### Superoxide dismutase assay

The SOD activity was evaluated by observing its ability to inhibit the photochemical reduction of nitro blue tetrazolium (NBT) according to Giannopolitis and Ries^46^ method. The optical density (OD) was measured at 560 nm. The reaction mixture was 50 mM of sodium phosphate buffer (pH 7.8), 13 mM of methionine, 2 mM of riboflavin, 75 mM of NBT, 100 mM of EDTA and 100 mL of enzyme extract (Supplementary Data 1).

### Ascorbate peroxidase assay

The activity of APX was quantified by monitoring the decrease in absorbance at 290 nm. Reaction mixture (1 mL) containing 50 mM of phosphate buffer (pH 7.6), 0.1 mM of Sodium-EDTA, 12 mM of H_2_O_2_, 0.25 mM of ascorbic acid and plant extract.

### Protein analysis

Protein contents of the chilli plants were determined by following Bradford^47^.

### Native-PAGE analysis of POD, CAT, SOD and APX

Expression of enzyme isoforms of Hybrid (HiR) and Padron (HS) pepper varieties were detected by discontinues native PAGE using a Mini-Protean II electrophoresis system^48^. Expression pattern of POD was determined by in-gel staining by the procedure of Abeles and Biles^49^. Total 80 µg protein of HiR and HS varieties at 48 h.p.i. and 72 h.p.i. of both inoculated and control was separated on the gel (5% stacking and 10% resolving) by applying a constant voltage of 200 V for 3 h. CAT isoforms were stained by a previously described protocol of Yang et al.^50^. Enzyme extracts of HiR and HS chilli pepper varieties at 48 h.p.i. of both inoculated and control were applied to gel (4% stacking and 7% resolving) for 8 h at 80 V. SOD isoforms were detected and stained by Beauchamp and Fridovich^51^ method. Extracts of HiR and HS varieties at 48 h.p.i. were applied to 4% stacking and 10% resolving for 3 h at 200 V. APX isoforms were stained by the Mittler and Zilinskas^52^ method. HiR and HS chilli varieties at 0 h.p.i. and 24 h.p.i. were selected for APX isoform analysis. 4% stacking gel and 10% resolving gel were used by applying a constant voltage of 100 V for 6 h.

### In-gel staining of isoforms

POD isoforms were stained by incubating the gel in 250 mL of acetate buffer containing 1 mL guaiacol and 400 μL of H_2_O_2_ until the visual observation of brown-orange bands on a clear background. CAT gel was stained by incubating in H_2_O_2_ for 15 min; then, the gel was washed for 15 min with distilled water. After washing the gel was incubated on a shaker in 1% potassium ferrocyanide and 1% ferric chloride for 15 min or until white bands on dark green background appeared. For staining the SOD gel, it was first incubated in a solution containing 50 mL of 50 mM potassium phosphate buffer (pH 7.8) and 0.078 g of 0.48 mM nitro blue tetrazolium in the dark for 20 min. Subsequently, the above solution was replaced by the same buffer containing 28 μM riboflavin and 28 mM TEMED for 15 min in the dark until clear SOD bands against the blue background appeared. For APX staining, gels were equilibrated with 100 mL 50 mM potassium phosphate buffer (pH 7.0) containing 0.07 g of 2 mM ascorbate for 30 min. Then gels were incubated in the above buffer, having 4 mM sodium ascorbate and 2 mM H_2_O_2_ for 20 min. Gels were then washed in the phosphate buffer and stained in 50 mM potassium phosphate buffer (pH 7.8), having 28 mM TEMED and 2 mM NBT, while shaking gently for 2–3 min until the appearance of clear bands on an intense blue background.

### Statistical analysis

All experiments were performed twice. The obtained data were pooled, and analysis of variance (ANOVA) was conducted. The figure’s values are mean ± SE (n = 3) compared with the Fisher LSD test using p ≤ 0.05. Graphs were made using a paired comparison on Origin statistical software 2021; 9.8.0.200 (Student Version) OriginLab Corporation, Northampton, MA, USA (https://www.originlab.com/index.aspx?go=PRODUCTS/OriginStudentVersion)^53^.

### Ethics approval and consent to participate

We all declare that manuscripts reporting studies do not involve any human participants, human data, or human tissue. So, it is not applicable.

### Complies with international, national and/or institutional guidelines

Experimental research and field studies on plants (either cultivated or wild), comply with relevant institutional, national, and international guidelines and legislation.

## Conclusions

Present results also suggest that increased levels of built-up antioxidant enzymes also decrease the scavenging potential of ROS produced under bacterial stress. Increased activity of peroxidase, catalase, and superoxide dismutase in chilli cultivars are associated with the plant resistance to bacterial spot disease and also are involved in the early response to is causal agent, whereas ascorbate peroxidase is less associated with stress in the early growth process. Furthermore, Isoforms synthesis confirmed that antioxidant enzymes are produced in many cell organelles due to the biotic stress and lead to resistance expression. Present investigations cover the expression of antioxidants as isoforms. In the future, the transcription expression of these enzymes must be further investigated. There is a need to explore the mechanistic details and factors that regulate antioxidant enzymes’ expression.

## Supplementary Information

Supplementary Information.
