# Supplementary material for: Mitigation of bacterial spot disease induced biotic stress in Capsicum annuum L. cultivars via antioxidant enzymes and isoforms
Source: Sci Rep. 2021 May 3;11:9445. doi: 10.1038/s41598-021-88797-1 (PMC8093210; doi:10.1038/s41598-021-88797-1)

Mitigation of Bacterial Spot Disease Induced Biotic Stress in *Capsicum annum* L. varieties via Antioxidant Enzymes and Isoforms

Musarrat Ramzan[1*], Sundas Sana[1], Nida Javed[1], Anis Ali Shah[2], Samina Ejaz[3], Waqas Nazir Malik[3], Nasim Ahmad Yasin[4], Saud Alamri[5], Manzer H. Siddiqui[5], Rahul Datta[6*], Shah Fahad[7,8*] Nazia Tahir[9,10], Ghulam Sabir Hussain[11], Niaz Ahmed[12], Muhammad Arif Ali[12], Subhan Danish[12*]

Supplementary data for gels:

POD image results of gel electrophoresis.

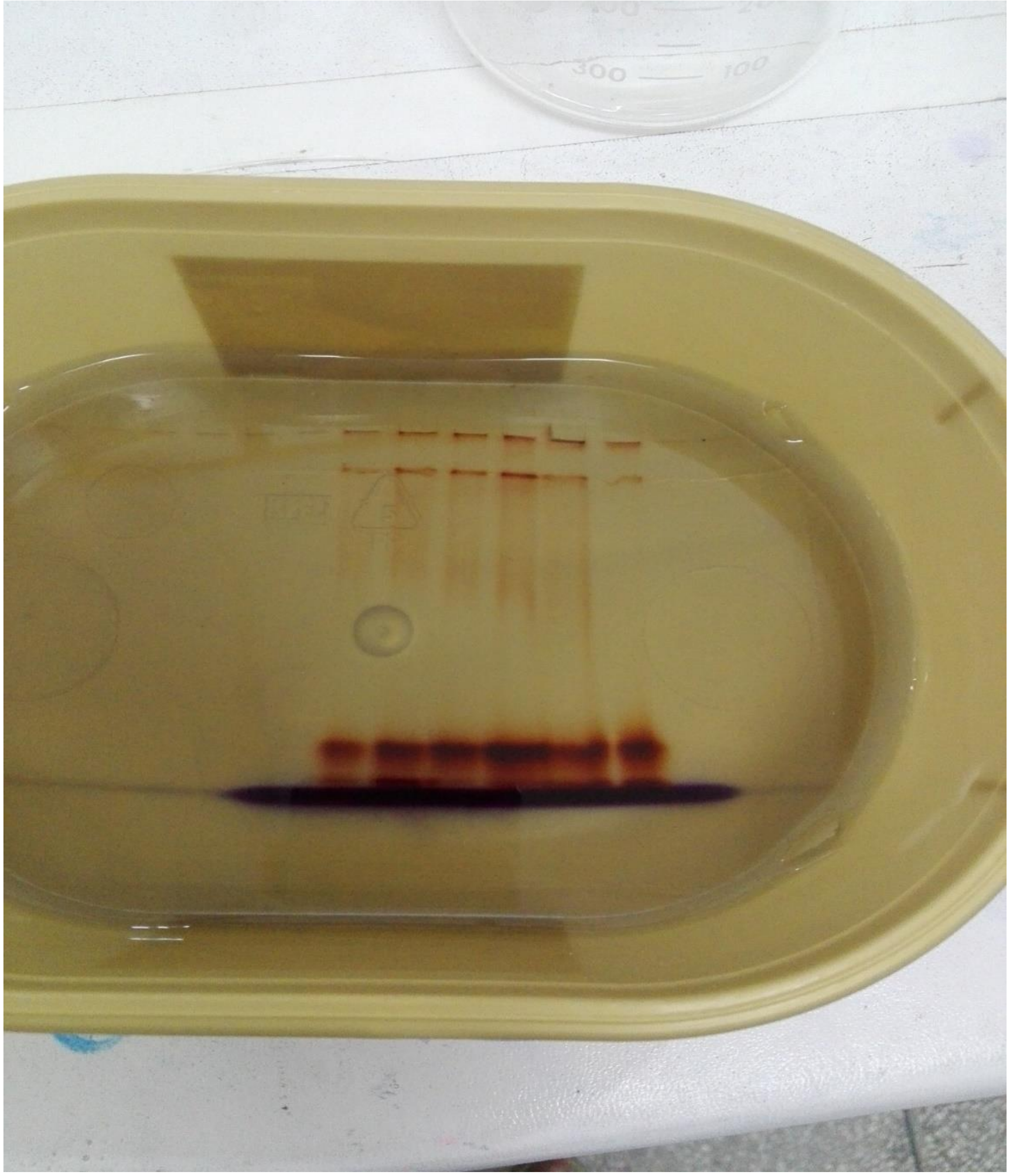

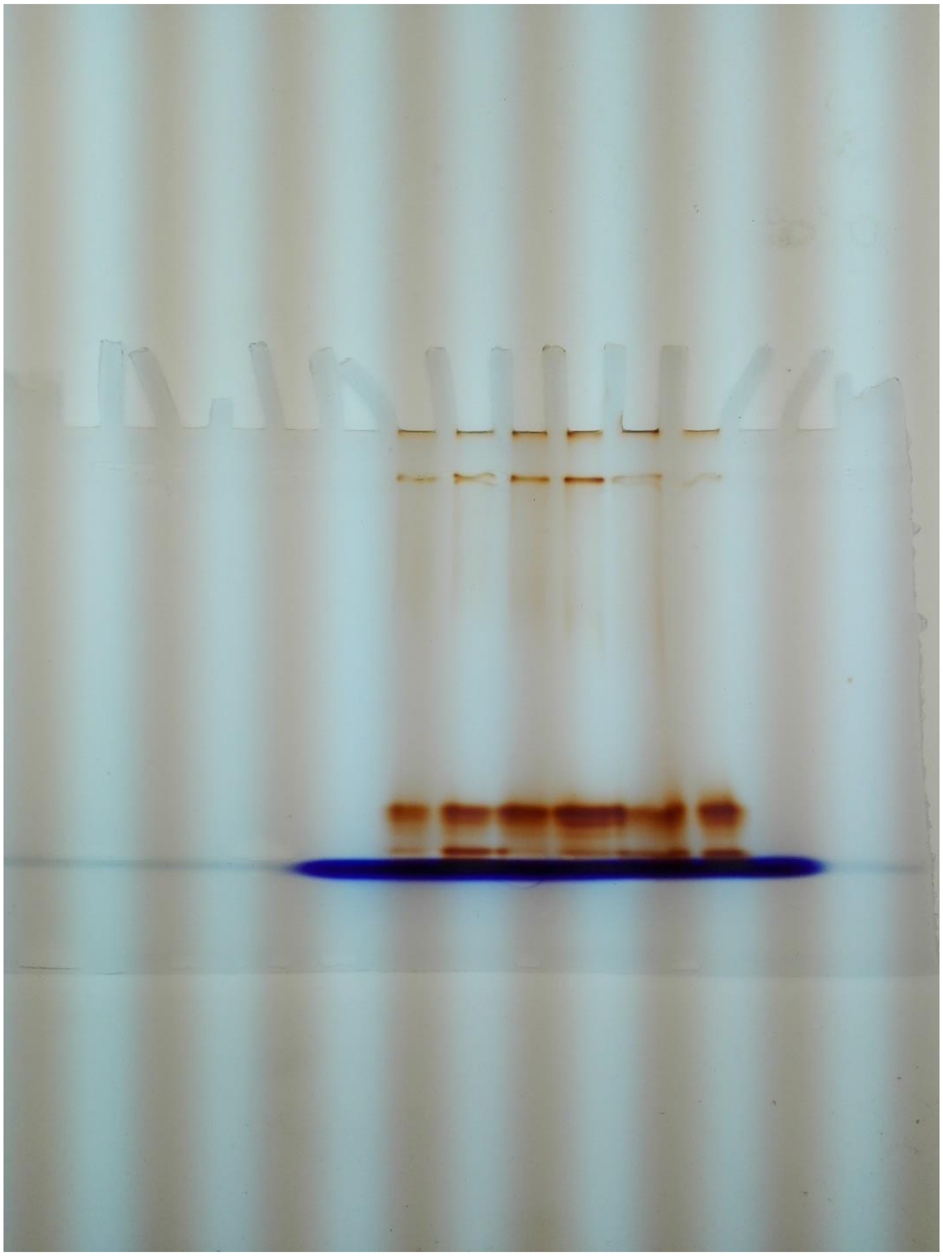

CAT image by gel electrophoresis:

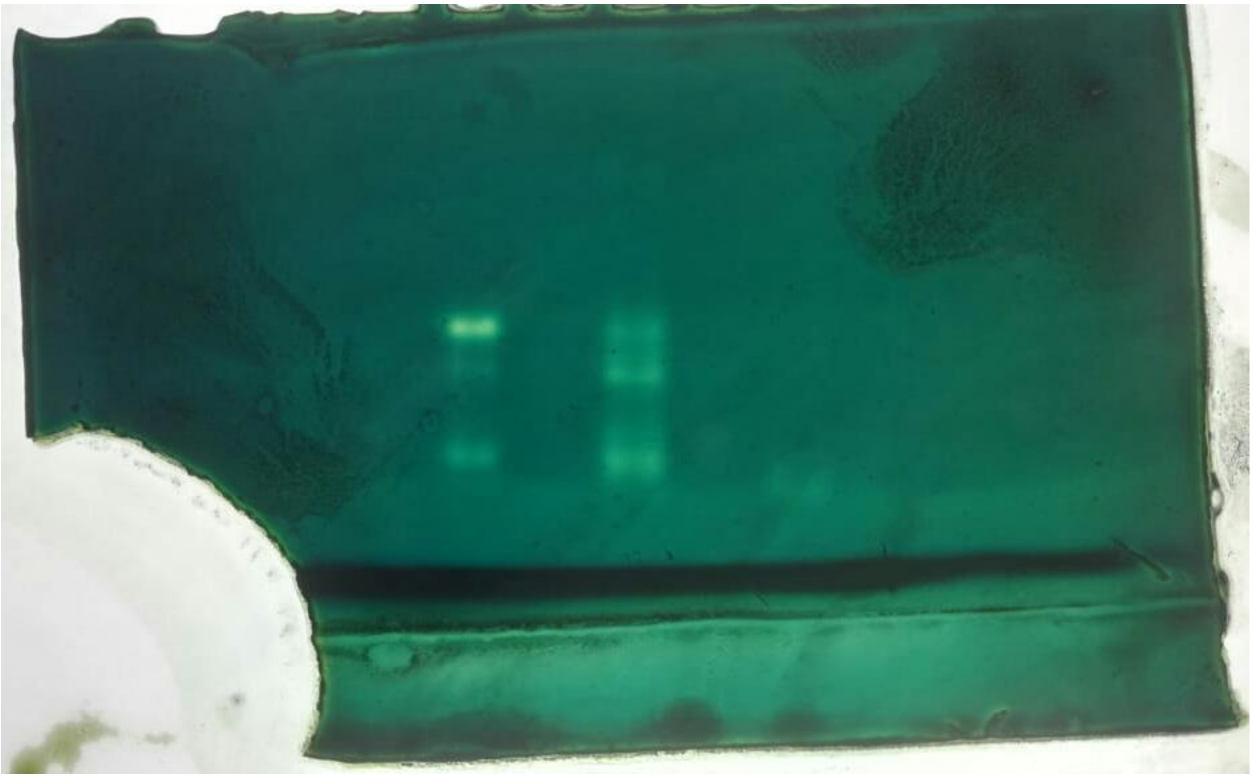

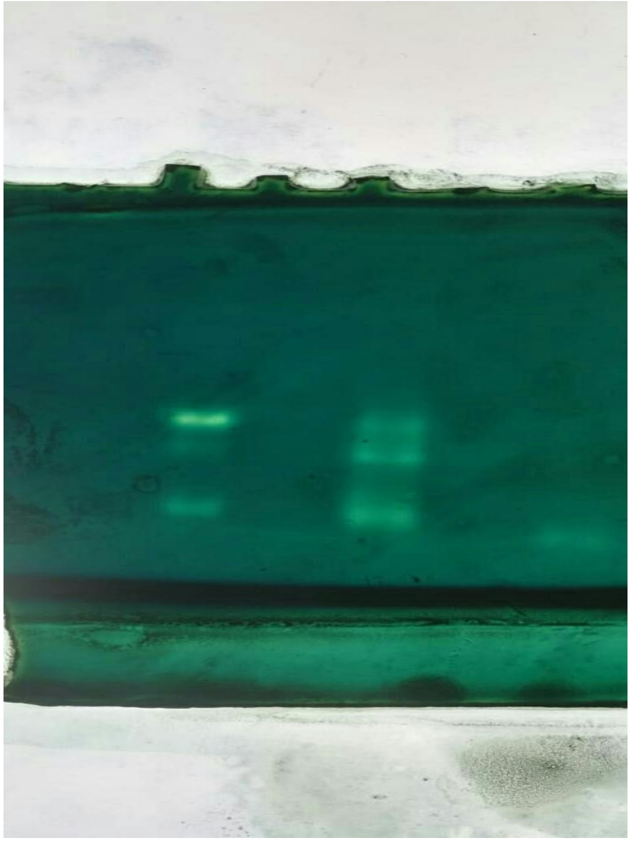

SOD image result by gel electrophoresis:

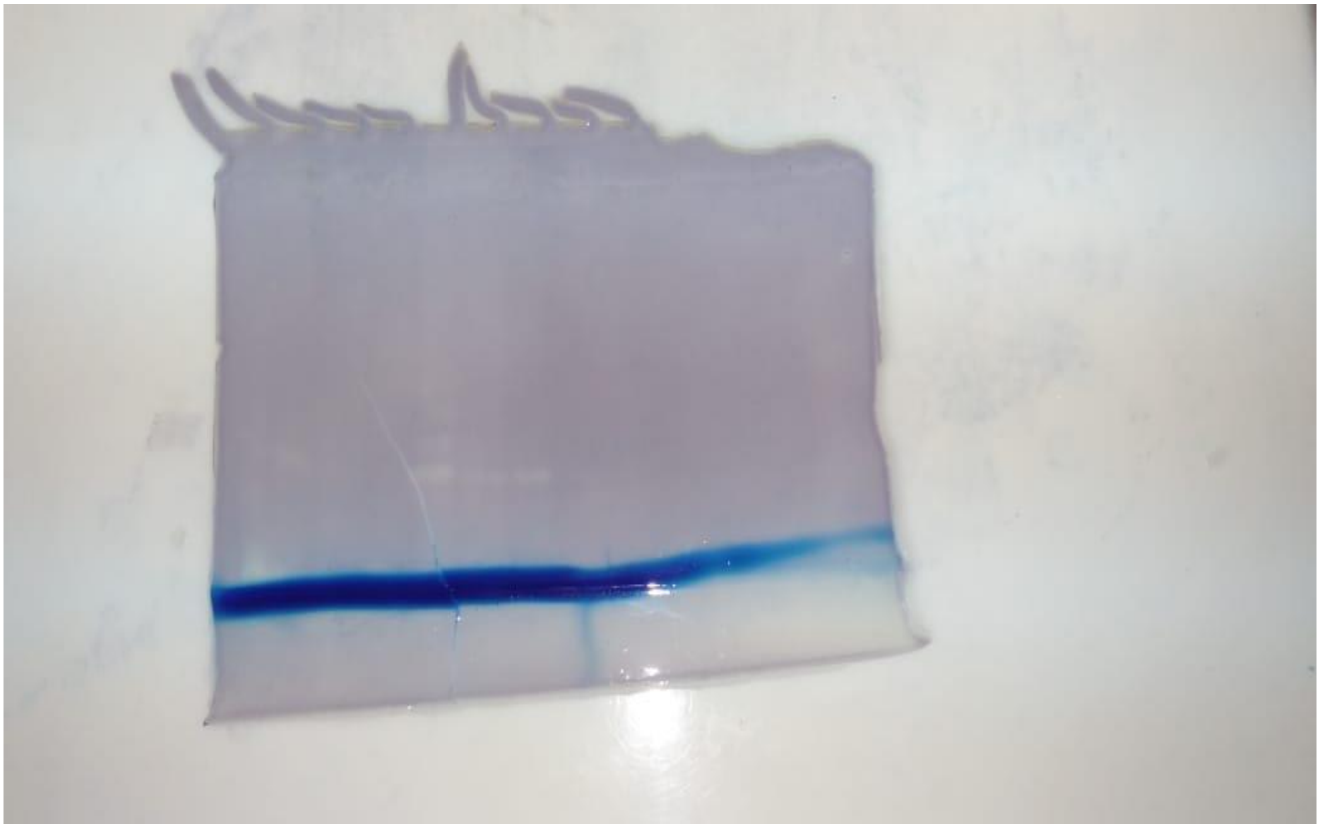

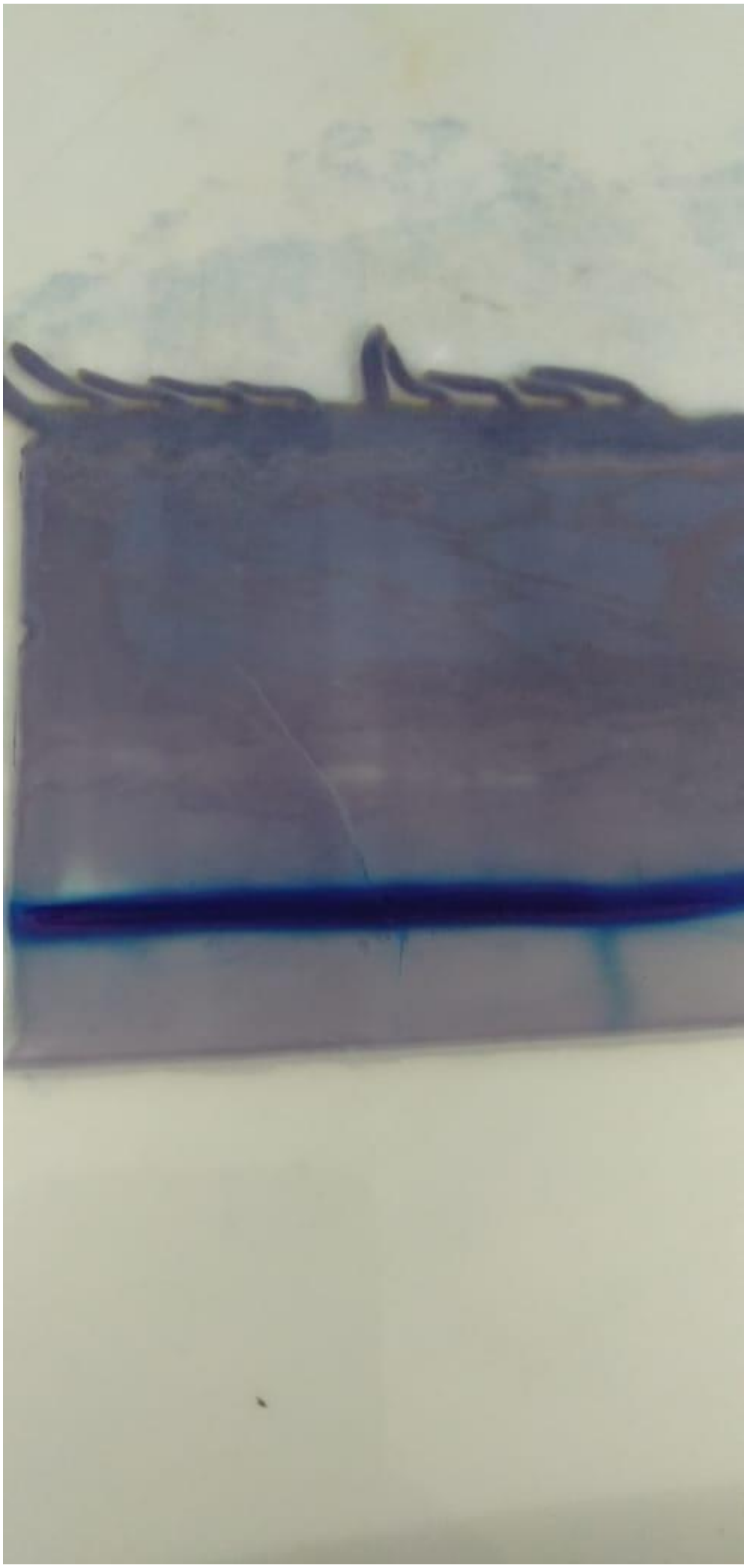

APX image result by gel electrophoresis:

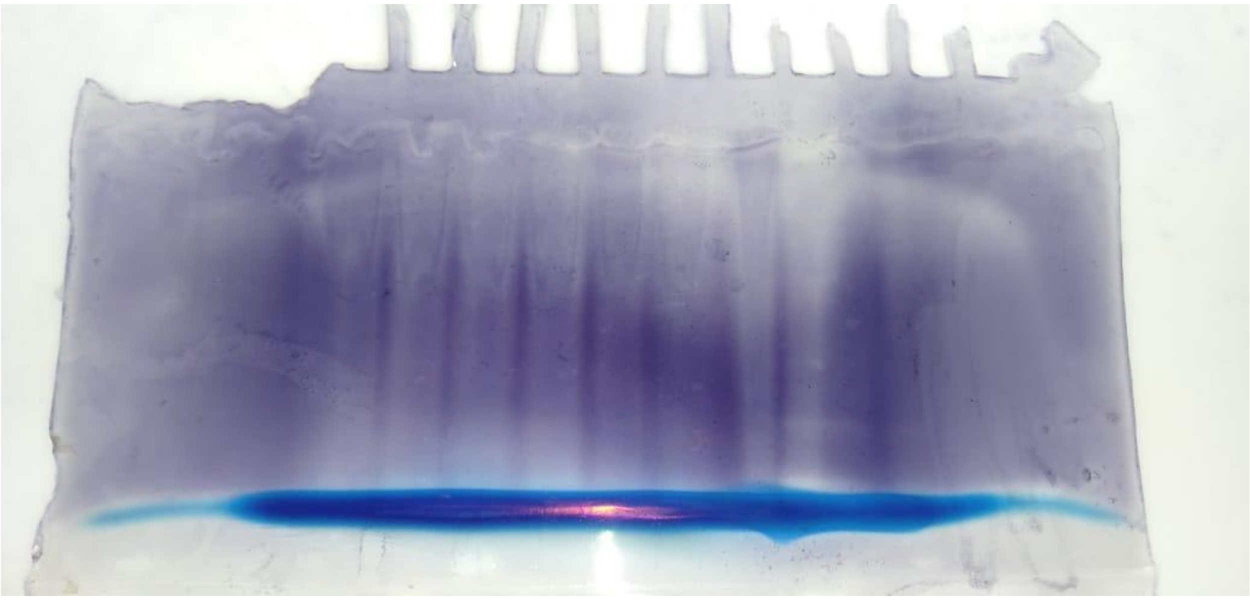

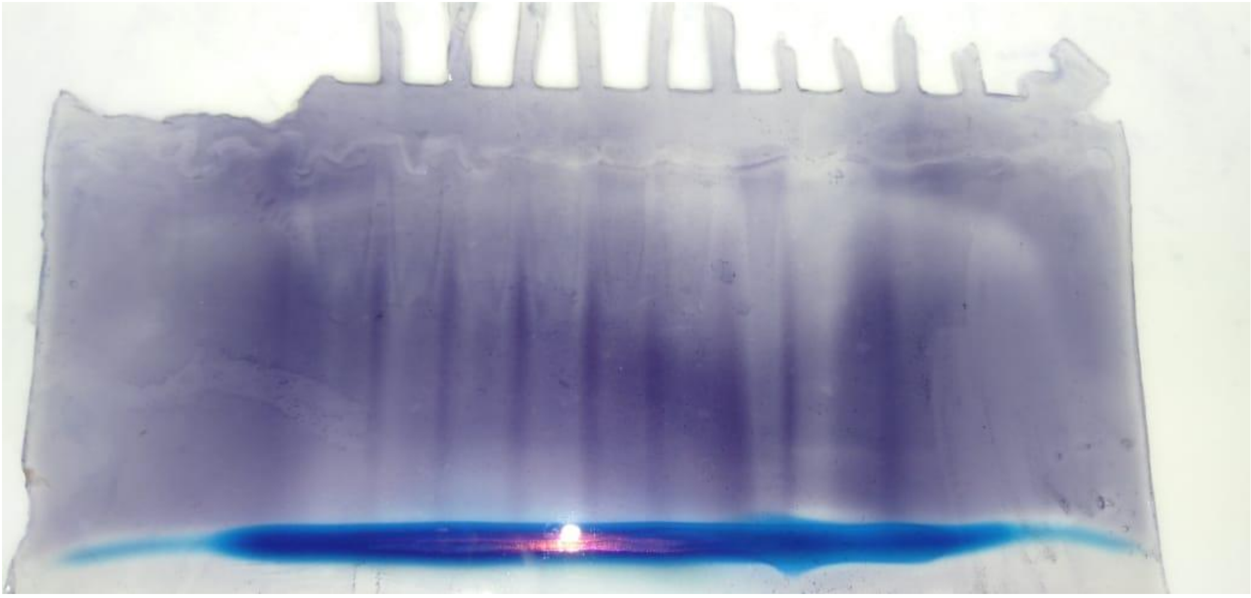

Supplement: Supplementary file 1 — Supplementary Information. [file 41598_2021_88797_MOESM1_ESM.pdf]
